# The Immune System in Tissue Environments Regaining Homeostasis after Injury: Is “Inflammation” Always Inflammation?

**DOI:** 10.1155/2016/2856213

**Published:** 2016-08-11

**Authors:** Onkar P. Kulkarni, Julia Lichtnekert, Hans-Joachim Anders, Shrikant R. Mulay

**Affiliations:** ^1^Department of Pharmacy, Birla Institute of Technology and Science Pilani, Hyderabad Campus, Hyderabad 500078, India; ^2^Medizinische Klinik und Poliklinik IV, Klinikum der Ludwig-Maximilians Universität München, 80336 Munich, Germany

## Abstract

Inflammation is a response to infections or tissue injuries. Inflammation was once defined by clinical signs, later by the presence of leukocytes, and nowadays by expression of “proinflammatory” cytokines and chemokines. But leukocytes and cytokines often have rather anti-inflammatory, proregenerative, and homeostatic effects. Is there a need to redefine “inflammation”? In this review, we discuss the functions of “inflammatory” mediators/regulators of the innate immune system that determine tissue environments to fulfill the need of the tissue while regaining homeostasis after injury.

## 1. Introduction 

Inflammation is one of the major danger control programs of tissue pathology conserved during evolution till date with a major aim to resolve the infection, repair the tissue damage, and regain the state of tissue homeostasis [1, 2]. It is a highly complex but still a very well-coordinated process, classically triggered by infection or tissue injury. Historically, “inflammation” was initially defined based on the clinical representations by Hippocrates as* calor*,* rubor*, tumor, and* dolor* [3]. This definition was challenged by the discovery of microscope in the 19th century, and the microscopic presence of leukocytes at the site of infection or injury was called “inflammation” since then [4]. However, this simplistic definition of “inflammation” does no longer hold true in the 21st century mainly because of the advancements in immunology and leukocyte biology in the last decade. We now know that leukocytes present numerous immunoregulatory phenotypes, for example, M2 macrophages, regulatory T and B cells, and fibrocytes, having anti-inflammatory functions. This implies that the presence of leukocytes observed by pathologists at sites of infection or injury does not necessarily indicate “inflammation,” at least without further characterizing their functional phenotypes. As such, we now define “inflammation” based on the presence of proinflammatory leukocyte phenotypes along with the expression of proinflammatory cytokines.

**Figure 1:**
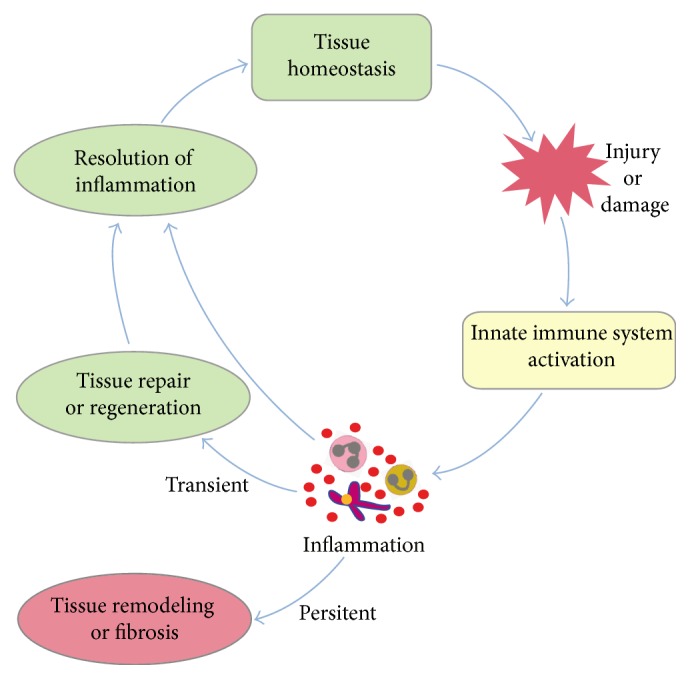
The role of the innate immune system in regaining tissue homeostasis. An injury disturbs the tissue homeostasis and activates the innate immune system leading to the recruitment of several immune cells at the site of injury. These immune cells secrete cytokines, growth factors, and enzymes to establish an inflammatory milieu. They also secrete anti-inflammatory and proregenerative cytokines to promote resolution of inflammation as well as tissue repair. A transient inflammation is often helpful to get rid of the cause of the tissue injury and return to homeostasis. However, an uncontrolled or persistent inflammation promotes tissue remodeling and fibrosis.

A successful inflammatory response eliminates the trigger followed by a resolution of inflammation and tissue repair by numerous anti-inflammatory cytokines as well as lipid mediators [5–8]. However, a persistent injurious trigger shifts the homeostatic set points fetching several changes in the initial inflammatory process (chronic inflammation), for example, replacement of neutrophils with macrophages and T cells and subsequent formation of granulomata or tertiary lymphoid tissues. In case these cellular effectors fail to control the injurious trigger, collateral tissue damage occurs [9–11]. Moreover, chronic inflammation can also arise as a result of autoimmune responses [9, 11]. Regardless of the cause, inflammation supposedly evolved to restore homeostasis. In this review, we discuss how different mediators of inflammation, in particular, of the innate immune system, set tissue environments to resolve inflammation and reinforce tissue repair, by promoting either regeneration or fibrosis in order to regain homeostasis after injury (Figure 1).

## 2. Resolution of Inflammation

An acute inflammatory response is followed by the resolution phase. The processes to return to tissue homeostasis, that is, catabasis [12], are governed by innate immune cells and specific mediators produced by them. These processes involve neutrophil apoptosis and their phagocytic removal via efferocytosis, clearance of proinflammatory dead cells and cytokines, and recruitment or phenotype switching of macrophages to anti-inflammatory phenotype [13]. Neutrophil-derived microparticles can also trigger the resolution of inflammation [13, 14]. Factors that mediate resolution include interleukin- (IL-) 10 and TGF-*β*, as well as lipid mediators, for example, lipoxins, resolvins, protectins, and maresins, collectively termed as specialized proresolving mediators (SPMs) [6, 15]. Within minutes after tissue injury prostaglandin and leukotriene synthesis from arachidonic acid metabolism occurs at the site of inflammation leading to the recruitment of neutrophils as a result of the chemotactic gradient, increased blood flow, and vascular permeability [16]. This is often followed by the class switching of lipid mediators, in which arachidonic acid metabolism switches from the production of leukotrienes to anti-inflammatory lipoxins, thus sending the “stop” signal to neutrophils recruitment and begins the end of the acute inflammatory response [17]. Lipoxins and resolvins stimulate the nonphlogistic phagocytosis of apoptotic neutrophils by monocyte-derived macrophages [18]. SPMs counterregulate the proinflammatory mediators and thus reduce the magnitude and duration of inflammation and tissue regeneration [12, 19].

Apart from limiting neutrophil recruitment, SPMs also help to increase natural killer (NK) cells mediated neutrophil apoptosis and subsequent efferocytosis by macrophages [20]. They potently inhibit the release of proinflammatory cytokines from the group 2 innate lymphoid cells (ILCs) [20] and increase IL-10 production by macrophages as well as induce M1 to M2 macrophage phenotype switch [21]. In addition to SPMs, the complement system also contributes to the resolution of inflammation by enhancing efferocytosis of apoptotic cells [22, 23]. The continuous phagocytosis of apoptotic cells, regulated by the mitochondrial membrane protein Ucp2 [24], stimulates monocytes to release IL-10 and TGF-*β* further promotes the switch toward an anti-inflammatory M2 macrophage phenotype [25, 26]. Recently, resolvin D1 has been demonstrated to trigger GPR32 to polarize macrophages toward the proresolving M2 phenotype [27]. Furthermore, IL-10 is an important cytokine with anti-inflammatory functions [28]. For example, in mouse models of acute kidney injury, IL-10 administration has a beneficial effect by inhibition of leukocyte infiltration and inflammatory renal cell death [29]. It also influences T cells by attenuating proliferation of CD4+ T cells and their cytokine production [30]. The tissue-resident dendritic cells (DCs) also promote the resolution of inflammation by producing pentraxin-3 (PTX3) which inhibits P-selectin on the vascular endothelial cells and thus inhibits immune cell recruitment to sites of injury [31–33]. Moreover, neutrophils released the prestored PTX3 in the early phase of acute myocardial infarction that bind to activated circulating platelets and dampen their proinflammatory response [34], whereas PTX3 also aggregated with histones and protected from histone-mediated endothelial cytotoxicity in sepsis [35, 36]. Furthermore, PTX3 suppressed complement dependent inflammation as well as reduced tumor infiltration by macrophages [37].

Group 3 ILCs gets activated and produces IL-22 after an intestinal epithelial injury suggesting that inflammation can override injury by promoting tissue regeneration [38]. Moreover, IL-22-producing ILCs prevented systemic inflammation during chronic diseases by promoting anatomical containment of lymphoid-resident commensal bacteria [39]. Similarly, the redox modification of high mobility group box 1 (HMGB1), a danger associated molecular pattern (DAMP) released after tissue injury as well as by macrophages and monocytes, regulated its proinflammatory functions during the resolution of inflammation and prevented excessive acetaminophen-induced hepatic injury [40, 41]. Together, immune cells, as well as mediators released by them, promote resolution of inflammation in order to reestablish the homeostasis after injury.

## 3. Tissue Regeneration and Repair

The immune system is instrumental in supplying growth factors and cytokine signals that orchestrate tissue repair. For example, the tissue-resident macrophages originated from yolk sac-derived erythromyeloid progenitors that possess the capacity to self-replenish [42, 43], while bone-marrow-derived circulating monocytes differentiate into tissue macrophages [44], but both are activated during injury. Blood-derived young monocytes/macrophages have enhanced remyelinating activity compared to old macrophages in the central nervous system [45]. Although M2 macrophages are the main driver of the resolution of inflammation, tissue repair, and scar formation, the M1 macrophages clear cellular debris in order to prevent the persistence of toxic and immunogenic material at the site of injury. Therefore, depletion of M1 macrophages resulted in impaired healing and regeneration after myocardial as well as skeletal muscle injuries [46, 47]. In addition, M1 macrophages also activated proliferative myogenesis via IL-6, TNF-*α*, and IL-1*β* whereas M2 macrophages supported myogenic differentiation via TGF-*β* production during skeletal muscle regeneration [47, 48].

Moreover, infiltrating eosinophils secreted IL-4 to induce proliferation of fibro/adipocyte progenitor cells, which promoted clearance of necrotic debris and skeletal muscle regeneration [49]. The CXCL12-CXCR4 pathway regulates the recruitment of progenitors, the unipotent proliferative cells with a capacity of self-renewal [50], at the site of injury [51]. Other mediators of the innate immune system that induced progenitor cells proliferation and regeneration include leukotriene C4, which activated radial glial cell proliferation and neurogenesis either upon or without an injury [52], oncomodulin derived from neutrophils, and macrophages which promoted the optic nerve regeneration [53, 54]. Furthermore, macrophages derived Wnt suppressed Notch signaling and thus regulated the fate of hepatic as well as renal progenitor cells after liver and kidney injury, respectively [55, 56]. Macrophage-derived Wnt7b also stimulated epithelial responses and, thus, regarded critical for kidney repair and regeneration [57]. In the acidic tissue environments after skin, liver, and lung injury and arterial thrombosis, neutrophils and macrophages derived PTX3 promoted remodeling of the fibrin-rich inflammatory matrix ensuring normal tissue repair [58]. In addition, the complement system is also instrumental in promoting tissue repair and regeneration by inducing growth factors as well as disposal of dead cells [23]. In particular, C3a and C5a activated NF-*κ*B/STAT-3 and enhanced hepatocyte regeneration after liver injury [59]. Recent reports showed that the delayed postinjury administration of C5a inhibited caspase-3 mediated neuron apoptosis leading to improved regeneration and functional recovery after murine spinal cord injury [60], as well as administration of C3a retina regeneration via STAT-3 activation in the progenitor cells present in the eye [61].

**Figure 2:**
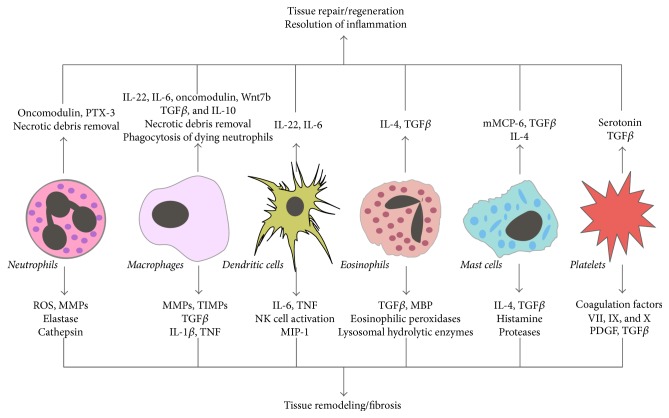
Mediators of innate immune system in regaining tissue homeostasis. Innate immune cells secrete several cytokines, growth factors, and enzymes, which promotes either resolution of inflammation and tissue repair/regeneration or tissue remodeling/fibrosis. PTX3: pentraxin 3, ROS: reactive oxygen species, IL: interleukin, TGF: transforming growth factor, MMP: matrix metalloproteinase, TIMP: tissue inhibitor of matrix metalloproteinase, TNF: tumor necrosis factor, MIP: macrophage inhibitory protein, MBP: major basic protein, mMCP: mouse mast cell protease, and PDGF: platelet-derived growth factor.

Apoptotic cells released after tissue injury promoted angiogenic properties of macrophages by releasing prostaglandin E2, which induced endothelial-derived progenitors to angiogenesis and vascular repair during skeletal muscle regeneration [62–64]. In addition, proliferation and differentiation of renal progenitor cells were also enhanced by the Toll-like receptor- (TLR-) 2-agonistic DAMPs released after tissue injury [65–67]. Several recent data suggests additional mechanisms of DAMP-driven tissue regeneration. For example, TLR4-agonistic DAMPs activated the interstitial mononuclear phagocytes to secrete a proregenerative cytokine IL-22 [68, 69] to promote tubular cell regeneration after injury by activating the JAK/STAT3 and ERK1/2 signaling pathway [68, 70]. Group 3 ILCs also produced IL-22 after an intestinal injury to promote the intestinal stem cell-mediated epithelial regeneration [71]. IL-22 mediated protection and regeneration were also observed in experimental models of hepatic, pancreatic, and thymic injuries [72–75]. In addition, a mast cell-specific tryptase, mouse mast cell protease (mMCP) 6, directly cleaves fibronectin and collagen IV and, therefore, suppressed scars and promoted functional recovery after spinal cord injury [76]. Platelets contributed to liver regeneration by secreting serotonin in mice as well as humans [77, 78]. Group 2 ILCs also promoted lung-tissue homeostasis after infection with influenza virus by producing a growth factor Amphiregulin [79]. After an injury to skeletal muscles, IL-33 recruited a special population of regulatory T cells (Tregs) to the injured muscles that produced Amphiregulin and improved the muscle repair [80–82]. Together, this illustrates that the innate immune system and its mediators do not only contribute to the immune injury but also to the immune-mediated repair or regeneration after injury as a part of a danger control response [10, 83] (Figure 2).

## 4. Tissue Remodeling and Fibrosis

The well-defined chronology of inflammatory events is essential for optimal repair. However, an overactivated immune response leads to tissue remodeling rather than tissue regeneration, which is clinically termed as tissue fibrosis. Fibrosis is characterized by excess deposition of extracellular matrix (ECM) due to the accumulation and activation of fibroblasts and myofibroblasts. Inflammatory cells of the immune system, as well as factors released by them, facilitate fibrosis. For example, tissue injury is always followed by altered vascular permeability to enhance the neutrophils recruitment to the site of injury. The delayed clearance of neutrophils from the site of injury further exacerbates the injury [84]. Neutrophils count is in fact used as a prognostic marker for cardiac remodeling [85]. Neutrophils are known to increase oxidative stress as well as release a number of enzymes like matrix metalloproteinases (MMPs), elastase, and cathepsins which contribute significantly to the process of fibrosis [86–88]. Apart from neutrophils, platelets can also respond to the state of infection or inflammation through activation of TLRs [89, 90]. The factors derived from platelets, for example, platelet-derived growth factor (PDGF), is a potent chemotactic agent, whereas TGF-*β* drives fibroblast proliferation and activation [91]. Moreover, the factors involved in coagulation can also contribute significantly to fibrosis, for example, factors VII, IX, and X [92–94]. The coagulation system and complement system are linked very closely, often involving a cross talk, to maintain the tissue homeostasis [23, 95]. For example, in the absence of C3, thrombin replaces the C3 dependent C5 convertase and directly cleaves C5 to generate the biologically active C5a [96], which induced fibrosis in lungs, liver, pancreas, and kidney after injury [97–100]. In addition to C5a, C3a has also been implicated in renal fibrosis [101].

Other cells of the innate immune system, for example, eosinophils and mast cells, also contribute to fibrosis. Eosinophils produce TGF-*β*, major basic protein (MBP)-1, eosinophilic peroxidase as well as granule proteins, and lysosomal hydrolytic enzymes which are implicated to be a part of fibrosis process [102]. Mast cells produce various proteases, vasoactive factors like histamine, cytokines, and TGF-*β* during the tissue injury and fibrosis [103]. IL-4, one of the major products of mast cell activation, contributes to the cardiac fibrosis [104]. Moreover, mast cell deficient mice are protected against pulmonary as well as cardiac fibrosis [105, 106]. Among the various immune cells, the macrophages are essential for efficient wound healing [107–111]. Macrophages are the main source of MMPs and tissue inhibitor of metalloproteinases (TIMPs) [109, 112, 113]. The balance between MMPs and TIMPs is crucial for maintaining the composition of ECM. Apart from MMPs and TIMPs, macrophages also contribute to the production of TGF-*β*, the most significant factor involved in fibrosis [114]. TGF-*β* regulates fibroblast activation, differentiation, and proliferation [115]. It also upregulates ECM genes and suppresses genes associated with MMPs, thus causing increased deposition of matrix. TGF-*β* promotes collagen synthesis and the expression of profibrotic genes such as type I collagen and connective tissue growth factor (CTGF) [15, 116]. Furthermore, TGF-*β* is also an anti-inflammatory factor; therefore, its early inhibition is associated with increased mortality, increased chemokine expression, and leukocyte infiltration while its inhibition during the resolution phase resulted in improved survival and reduced tissue fibrosis [117–122]. Activated macrophages induce production of various cytokines and factors like interleukins (e.g., IL-1*β*) and TNF-*α*, which drive further inflammation and fibrosis by enhancing ECM production as well as upregulating expression of TGF-*β* [123, 124]. Depletion of macrophages during the early phase of tissue injury ameliorated fibrosis, while delayed depletion of macrophages during the resolution phase exaggerated fibrosis with persistence of profibrotic cellular and matrix components [110, 111, 125]. Although recent studies demonstrated an association between macrophages derived PTX3 and tissue fibrosis in nonalcoholic fatty liver disease as well as in lung fibrosis, whether PTX3 causes fibrosis or not is still unclear [126, 127].

Along with macrophages, DCs are the primary determinants of the cytokine and chemokine milieu during fibrogenesis [128, 129]. For example, IL-6 and TNF-*α* produced by DCs have pleiotropic effects on liver fibrosis [128]. Furthermore, they also activated NK cells to produce TNF-*α* and, therefore, elevate the inflammatory environment in fibrotic livers [128]. Group 3 ILCs promoted bleomycin-induced pulmonary fibrosis by secreting IL-17 [130, 131], whereas IL-25 induced expansion of the group 2 ILCs within the lungs, which promoted pulmonary fibrosis via IL-13 dependent mechanism [132]. Together, the innate immune system and its mediators contribute to tissue remodeling and fibrosis (Figure 2).

## 5. Conclusions and Future Perspectives

Maintaining tissue morphology is essential to maintain tissue function, that is, homeostasis. An injury or damage affects the structural integrity of the tissue implying a loss of tissue function, and, therefore, the structural and functional recovery, that is, regaining homeostasis after injury, is the ultimate goal. The inflammatory mediators of the innate immune system are important regulators of tissue homeostasis. They modulate tissue environments at all phases of the homeostatic imbalance, for example, promotion as well as the resolution of inflammation, tissue regeneration, and tissue remodeling/fibrosis.

Our understanding of inflammation biology has increased over the last few decades and has gone far beyond the basic concept of inflammation that was originally introduced by Hippocrates. The advancements in microscopic as well as flow associated cell sorting (FACS) techniques have allowed us to understand and redefine the “inflammation.” This progress has also raised several questions, for example, what are the molecules or signals that regulate the function of the innate immune cells, what are the critical mechanisms that regulate the balance of different populations of these cells in the specific phase after injury, and how to modulate the behavior as well as balance of these cells in each phase after injury to enhance tissue regeneration and reduce fibrosis. The advancements in the new genomic technologies such as CRISPR-Cas9 have transformed the field of immunology research and will certainly speed up novel discoveries in regulatory and signaling components of inflammation biology.

The newly obtained knowledge will translate into novel therapeutic strategies for inflammatory diseases. For example, recent studies have identified proinflammatory and proregenerative potential of a cytokine IL-22 and a regulatory oncoprotein murine double minute- (MDM-) 2 in the pathogenesis of ischemic renal injury (IRI) and have demonstrated the therapeutic potential of recombinant IL-22 and MDM-2 inhibitor, nutlin-3a, in IRI and other inflammatory diseases [68, 133–135]. Moreover, other studies have identified a pattern recognition molecule PTX3 as a potential target for therapeutic manipulation in damaged tissues as well as a variety of diseases [37, 58]. Therefore, in-depth understanding of the functions of inflammatory cells as well as mediators of inflammation will be instrumental in the identification of novel therapeutic targets and treatment strategies for several inflammatory diseases. As written by a Scottish surgeon in 1974 “Inflammation in itself is not to be considered as a disease but as a salutary operation consequent to some violence or some disease” [136].
